# Mean Annual Temperature, Soil Organic Matter and Phyllospheric Bacterial Diversity Shape Biomass of Dominant Species Along a Degradation Gradient in Alpine Steppes: A Case Study from the Qinghai–Tibet Plateau

**DOI:** 10.3390/microorganisms13122787

**Published:** 2025-12-07

**Authors:** Kaifu Zheng, Xin Jin, Jingjing Li, Guangxin Lu

**Affiliations:** College of Agriculture and Animal Husbandry, Qinghai University, Xining 810016, China; zhengkf@qhu.edu.cn (K.Z.); 18894310895@163.com (X.J.); lijingjing1879705@163.com (J.L.)

**Keywords:** high-altitude grasslands, *Stipa purpurea*, grassland deterioration, microbial resistance potential

## Abstract

The structure and function of alpine steppes are maintained largely by dominant species, which in turn determine the productivity and stability of plant communities. Nutrient acquisition and stress regulation may, to some extent, be mediated by phyllospheric microbiota at the interface of plants with the atmosphere, and phyllospheric microbes are capable of amplifying and transmitting vegetation responses to degradation. Previous research has mainly addressed climate, soil, vegetation and soil microbiota or has assessed phyllosphere communities as a whole, thereby overlooking the specific responses of phyllospheric bacteria associated with the vegetation-dominant species *Stipa purpurea* along gradients of vegetation degradation in alpine steppes. In this study, we characterised vegetation degradation at the community level (from non-degraded to severely degraded grasslands) and quantified associated changes in the dominant species *Stipa purpurea* (cover, height and aboveground biomass) and its phyllospheric bacterial communities, in order to elucidate response patterns within the coupled system of host plants, phyllosphere microbiota, climate (mean annual temperature and precipitation) and soil physicochemical properties. Compared with non-degraded (ND) grasslands, degraded sites had a 22.6% lower mean annual temperature (MAT) and reductions in total nitrogen, nitrate nitrogen, organic matter (OM) and soil quality index (SQI) of 49.4%, 55.6%, 46.8% and 47.6%, respectively. Plant community cover and the aboveground biomass of dominant species declined significantly with increasing degradation. Along the vegetation-degradation gradient from non-degraded to severely degraded alpine steppes, microbial source-tracking analysis of the phyllosphere of the dominant species *Stipa purpurea* revealed a sharp decline in the contribution of phyllospheric bacterial sources. Estimated contributions from non-degraded sites to lightly, moderately and severely degraded sites were 95.68%, 62.21% and 6.89%, respectively, whereas contributions from lightly to moderately degraded and from moderately to severely degraded sites were 34.89% and 16.47%, respectively. Bacterial richness increased significantly, and β diversity diverged under severe degradation (PERMANOVA, F = 5.48, *p* < 0.01). From light to moderate degradation, biomass and relative cover of the dominant species decreased significantly, while the phyllosphere bacterial community appeared more strongly influenced by the host than by environmental deterioration; the community microbial turnover index (CMTB) and microbial resistance potential increased slightly but non-significantly (*p* > 0.05). Under severe degradation, worsening soil conditions and hydrothermal regimes exerted a stronger influence than the host, and CMTB and microbial resistance potential decreased by 6.5% and 34.1%, respectively (*p* < 0.05). Random-forest analysis indicated that climate, soil, phyllosphere diversity and microbial resistance jointly accounted for 42.1% of the variation in constructive-species biomass (R^2^ = 0.42, *p* < 0.01), with the remaining variation likely driven by unmeasured biotic and abiotic factors. Soil contributed the most (21.73%), followed by phyllosphere diversity (9.87%) and climate (8.62%), whereas microbial resistance had a minor effect (1.86%). Specifically, soil organic matter (OM) was positively correlated with biomass, whereas richness, beta diversity and MAT were negatively correlated (*p* < 0.05). Taken together, our results suggest that under ongoing warming on the Qinghai–Tibet Plateau, management of alpine steppes should prioritise grasslands in the early stages of degradation. In these systems, higher soil organic matter is associated with greater phyllospheric microbial resistance potential and increased biomass of *Stipa purpurea*, which may help stabilise this dominant species and slow further vegetation degradation.

## 1. Introduction

Alpine grasslands, as integral components of cold region ecosystems, are widely recognised to serve as essential reservoirs for regional water conservation [1] and as central platforms for carbon and nitrogen cycling and biodiversity maintenance [2,3]. Within this broad category, alpine steppes provide irreplaceable support for pastoral production at high elevations [4], soil stability and surface processes, and landscape connectivity [5,6]. Over recent decades, however, alpine steppe degradation of varying severity has been documented widely under the combined pressures of climate warming and intensified human activity. Consequent reductions in vegetation cover, weakened soil structure and diminished ecological functions constrain the long-term sustainability of pastoral systems and elevate regional environmental risks [1,7,8,9].

At the scale of vegetation communities, dominant species serve as pivotal hubs sustaining community structure and function. Their abundance and vitality are widely employed as sensitive biological indicators of grassland degradation [10,11]. Phyllospheric microorganisms, located at the interface between the plant, the atmosphere and the soil, contribute directly to host nutrient acquisition [12], microenvironmental regulation and stress mitigation [13]. Because their community assembly is shaped jointly by host traits and external pressures, phyllosphere microbiota respond markedly to environmental change [14]. Compared with fungi, phyllospheric bacteria show higher community-level dispersal rates, greater taxonomic and functional diversity, faster turnover and stronger host dependence [15]. As a result, phyllosphere bacterial communities are able to capture and transmit vegetation-degradation signals more rapidly and at finer spatial scales [16]. Nevertheless, research on alpine steppe degradation has focused mainly on climate, vegetation, soil and soil microbiota and has rarely examined phyllosphere microbes in relation to specific dominant host species [17,18]. Systematic evidence is still lacking for how dominant species and their phyllosphere bacteria correspond along degradation gradients and how the relative importance of host filtering vs. environmental filtering shifts across successive stages of degradation. Clarifying these coupled dynamics among the host, the phyllosphere and climate–soil factors improve our understanding of biomass variation in the dominant species *Stipa purpurea* along degradation gradients in alpine steppes.

To fill the knowledge gaps identified above, we selected four alpine steppe sites representing a degradation gradient: non-degraded (ND), lightly degraded (LD), moderately degraded (MD) and severely degraded (SD). To minimise host effects on the phyllospheric bacterial communities, we focused exclusively on the dominant species *Stipa purpurea*, the dominant tussock grass shaping alpine steppe communities. Within grassland succession, the cover and biomass of *S. purpurea* largely determine ecosystem functioning and plant diversity [19], and its decline is widely recognised as an early warning indicator of steppe degradation [20,21,22]. By integrating quadrat vegetation surveys with high throughput sequencing, we addressed four questions: (1) How do climate, soil properties, plant community attributes and *S. purpurea* biomass change along the degradation gradient? (2) How do the alpha and beta diversity of *S. purpurea* phyllosphere bacteria vary with degradation, and which factors drive these patterns? (3) How does the resistance of the phyllosphere bacterial community to environmental change differ among degradation stages? (4) Which variables best explain shifts in *S. purpurea* biomass? By coupling a constructive host species with its phyllosphere microbiota across a well-defined degradation gradient, this study aims to quantify how these variables jointly relate to variation in *S. purpurea* biomass in alpine steppes.

## 2. Materials and Methods

### 2.1. Description of the Study Area and Selection of Host Plants for Phyllosphere Sampling

**Figure 1:**
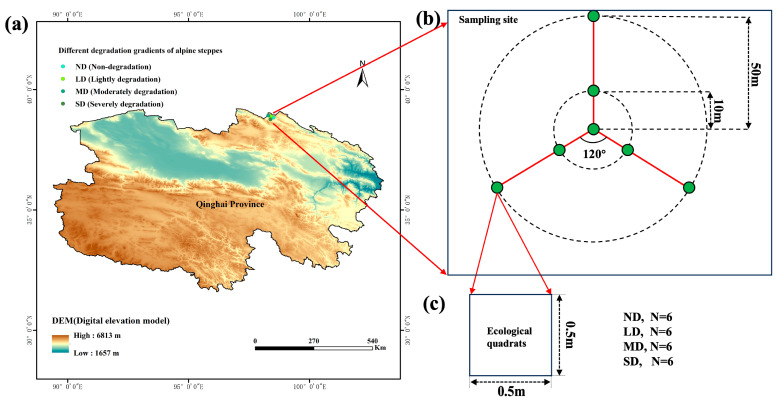
Experimental site, sampling site and ecological quadrats. (**a**) Geographic location of alpine steppe plots on the Qinghai–Tibet Plateau. (**b**) Arrangement of sampling site. (**c**) Arrangement of three 0.5 m × 0.5 m quadrats within each site.

Four representative alpine steppe sites were selected along the northern fringe of the Qinghai–Tibetan Plateau in China for comparative analysis (Figure 1a). Based on total community cover, total aboveground biomass, soil organic matter, and the cover and aboveground biomass of the dominant species *Stipa purpurea* (a recognised indicator of alpine steppe degradation [19]), sites were assigned to one of four degradation classes: non-degraded, lightly degraded, moderately degraded and severely degraded (Table 1). Geographic coordinates for all sites are listed in Table 1, and their spatial distribution is illustrated in Figure 1a. The four alpine steppe sites representing the non-degraded, lightly degraded, moderately degraded and severely degraded grasslands are separated by inter-site distances ranging from 5.47 km to 13.07 km. Along the degradation gradient, the non-degraded (ND) alpine steppe corresponds to Cryosols with a silt loam texture, the lightly degraded (LD) steppe to Cambisols with a loam texture, the moderately degraded (MD) steppe to Gleysols with a sandy loam texture and the severely degraded (SD) steppe to Regosols with a loamy sand texture (Supplementary Table S1). Climatic data were sourced from decadal (2013–2023) gridded datasets at 1 km spatial resolution provided by the National Earth System Science Data Center (http://loess.geodata.cn; accessed 14 January 2024). Mean annual temperature and precipitation were extracted in ArcGIS (version 10.8) by overlaying the 1 km climate rasters with site coordinates, yielding mean annual temperatures of −4.01 to −1.85 °C and mean annual precipitation of 186.70 to 420.99 mm.

The vegetation of alpine steppes is dominated by *Stipa purpurea*, *Leymus secalinus*, *Carex tristachya* and *Oxytropis ochrocephala*. *S. purpurea* was therefore selected as the sole host for phyllospheric bacterial community sampling to track microbial responses along the degradation gradient. This decision provides several ecological and methodological advantages. Firstly, as the quintessential dominant species of the Qinghai–Tibet alpine steppe, *S. purpurea* occurs across all degradation stages, and its leaf morphology and physiology are relatively conserved within populations. Using a single host species minimises taxonomic variation among plants in microbial community structure, thereby increasing the signal-to-noise ratio in community response analyses [23]. Secondly, being a keystone species that maintains grassland structure, material cycling and energy flow, *S. purpurea* is highly sensitive to environmental stress and degradation. Its physiological status and microbial symbioses have been documented as early warning signals of alpine steppe degradation [19], reflecting shifts in soil organic carbon, nitrogen cycling and microbial network stability [18]. Furthermore, contemporary studies that assess environmental controls on phyllosphere microbiota commonly adopt a single-host design to eliminate host species effects on community assembly [24]. Our selection of *S. purpurea* therefore conforms to global methodological standards while providing strong ecological relevance for alpine steppe restoration research.

### 2.2. Plot Layout and Vegetation Survey, Phyllosphere Sampling and Preservation, and Soil Collection and Physicochemical Measurements

All field sampling was conducted in August 2023. Four representative alpine steppe sites corresponding to a degradation gradient—non-degraded (ND), lightly degraded (LD), moderately degraded (MD) and severely degraded (SD)—were selected along the northern fringe of the Qinghai–Tibet Plateau in China for comparative analysis (Figure 1a). The distances between sites ranged from 5.47 km to 13.07 km. Within each alpine steppe site, we established a central reference point and laid out three transects radiating from this point at 120°. On each transect, two sampling points were positioned at 10 m and 50 m from the central point, yielding six sampling points per site (Figure 1b). The distance between sampling points within a site ranged from 10 m to 103.92 m. At each sampling point, three 50 cm × 50 cm quadrats were randomly placed at least 1 m apart (Figure 1c). Within each quadrat, we surveyed the entire plant assemblage and recorded total plant community cover, as well as the percentage canopy cover of the dominant species Stipa purpurea within the 50 cm × 50 cm frame. Aboveground biomass of both the whole plant community and S. purpurea was harvested and weighed. For each sampling point, measurements from the three quadrats were averaged to obtain a single point-level value, and the six point-level values per degradation level were then used as independent replicates in subsequent statistical analyses.

Phyllosphere samples of *S. purpurea* were collected immediately after the vegetation survey. Within each quadrat, 8 to 10 visibly healthy tillers free of lesions were randomly selected, and their leaf blades (approximately 200 cm^2^ in total area) were excised with scissors sterilised with ethanol while the operator wore sterile gloves. Leaves were transferred on site to sterile 250 mL centrifuge bottles prefilled with 100 mL sterile PBS (0.1% Tween 80, pH 7.0) that were uniquely barcoded to match the quadrat. The bottles were gently inverted for 30 s to dislodge epiphytic microbes, placed in a mobile freezer at −20 °C, transported to the laboratory and stored at −80 °C until DNA extraction.

After phyllosphere collection, bulk soil samples were collected for physicochemical analysis. At each sampling point, three 50 cm × 50 cm quadrats were used as the soil sampling frame. Within each quadrat, three soil cores (0–20 cm depth) were taken along the diagonal using a sterile soil auger (Eijkelkamp Agrisearch Equipment, Giesbeek, The Netherlands). The nine cores from the three quadrats at each sampling point were thoroughly homogenised to form one composite bulk soil sample. Thus, six composite soil samples were obtained for each degradation level. Each composite sample was sealed in sterile zip-lock bags, labelled, kept in a mobile freezer at −20 °C during transport and then stored at −20 °C until laboratory analyses. In the laboratory, soils were thawed, gently homogenised, air-dried at room temperature and passed through a 1 mm sieve to remove visible fine roots, stones and organic debris prior to physicochemical measurements.

Soil physicochemical analyses: Total nitrogen (TN) was analysed via the semi-micro Kjeldahl method [25]. Total phosphorus (TP) was determined by the continuous-flow ammonium molybdate spectrophotometric method after acid digestion, and available phosphorus (AP) was extracted with 0.5 mol L^−1^ NaHCO_3_ (pH 8.5; Olsen method) and measured with the same colourimetric procedure. Available potassium (AK) was extracted with 1 mol L^−1^ NH_4_OAc (pH 7.0) and determined by flame photometry [26]. Organic matter (OM) was quantified using the potassium dichromate–sulfuric acid external heating method [27]. Mineral nitrogen was extracted with 2 mol L^−1^ KCl (soil–solution = 1:5); nitrate nitrogen (NO_3_^−^–N) in the extracts was determined by ultraviolet spectrophotometry [28], and ammonium nitrogen (NH_4_^+^–N) by the indophenol blue colorimetric method [29]. In situ soil electrical conductivity (EC) and soil moisture content (SMC) at 0–20 cm depth were measured with a three-parameter sensor (TDR-350, Spectrum Technologies, Inc., Aurora, IL, USA).).

### 2.3. Processing, Sequencing and Bioinformatic Analysis of Phyllosphere Bacterial Communities

#### 2.3.1. Elution of Microbes from Phyllosphere Samples

More than 5 g of fresh leaf material were placed into a sterile 250 mL Erlenmeyer flask containing 100 mL of phosphate-buffered saline (PBS; 0.02 M, pH 7.0) and 100 μL of Tween 80. The suspension was shaken on a rotary platform for 30 min and subsequently subjected to sonication for 4 min. The resulting wash solution was passed through a 0.22 μm membrane filter to capture phyllosphere microbes. This extraction procedure was repeated twice for each sample. Filters were then kept at −80 °C until DNA extraction.

#### 2.3.2. DNA Extraction and Quality Assessment

Microbial DNA was extracted from the filters using the FastDNA SPIN Kit for Soil (MP Biomedicals, Solon, OH, USA) following the manufacturer’s protocol. DNA yield and purity were assessed with a NanoDrop 2000 spectrophotometer (Thermo Fisher Scientific, Waltham, MA, USA), and DNA integrity was checked by electrophoresis on a 1% agarose gel run at 5 V cm^−1^ for 20 min. In total, 24 independent phyllosphere samples (six per degradation level: ND, LD, MD and SD) were processed for DNA extraction and subsequent sequencing. To monitor potential contamination in these low-biomass phyllosphere samples, DNA extraction blanks and PCR no-template controls were processed in parallel; these negative controls produced no visible amplicons and no usable sequence data and were therefore excluded from subsequent analyses.

#### 2.3.3. PCR Amplification of the 16S rRNA Gene

To capture broad phyllosphere bacterial diversity in alpine grasslands, we used universal primer pairs that are widely recommended for phyllosphere studies [30,31]. This approach was chosen to (i) maximise detection of rare and as-yet-undescribed taxa and (ii) avoid the strong taxonomic bias that genus-specific primers may introduce in broad community surveys. The V4 region of the bacterial 16S rRNA gene was amplified using primers 515F (5′ GTGYCAGCMGCCGCGGTAA 3′) and 806R (5′ GGACTACHVGGGTWTCTAAT 3′) [30].

#### 2.3.4. Verification, Purification and Sequencing of PCR Products

PCR products were first inspected on 2% agarose gel. Bands of the expected size and adequate intensity were quantified with a QuantiFluor ST fluorometer (Promega, Madison, WI, USA). Target bands were excised and purified using the AxyPrep DNA Gel Extraction Kit (Axygen Biosciences, Union City, CA, USA) according to the manufacturer’s instructions. Purified amplicons were pooled, transported on dry ice to Lingen Biotechnology (Shanghai, China) for library preparation and sequenced on an Illumina platform using 300 bp paired-end reads.

#### 2.3.5. Sequence Quality Control and OTU Clustering

Raw paired-end reads were first filtered with fastp (v.0.20.0; https://github.com/OpenGene/fastp accessed 14 January 2024) [32] to remove low-quality sequences. The retained read pairs were then merged using FLASH (v.1.2.7; https://ccb.jhu.edu/software/FLASH/ accessed 14 January 2024) [33] with the following criteria: trimming from the 3′ end with a 50 bp sliding window whenever the mean quality score dropped below 20; discarding reads shorter than 50 bp or containing ambiguous bases (N); merging read pairs with an overlap of at least 10 bp and a maximum mismatch ratio of 0.2; requiring exact matches for barcodes and allowing up to two mismatches in primer regions during demultiplexing; and correcting sequence orientation after demultiplexing.

#### 2.3.6. OTU Assignment and Taxonomic Annotation

High-quality sequences were grouped into operational taxonomic units (OTUs) at a 97% similarity threshold using the UPARSE pipeline [34,35]. Representative sequences from each OTU were taxonomically assigned with the RDP Classifier (v.2.2) trained on the RDP 16S rRNA reference training set [36]. The resulting OTU table was used in subsequent statistical analyses.

### 2.4. Statistical Analysis

Community cover, total biomass, *S. purpurea* cover, *S. purpurea* height and *S. purpurea* biomass were assessed across degradation classes. The residuals were examined for normality with the Shapiro–Wilk test (stats package), and variance homogeneity was checked with Levene’s test (car package). If both assumptions were met, a one-way ANOVA was carried out. When the overall test was significant (two-tailed, α = 0.05), pairwise differences were evaluated with Tukey HSD (agricolae package) to control the family-wise error rate. When residuals were approximately normal but variances were unequal, Welch one-way ANOVA was applied, followed—if the overall effect was significant—by Games-Howell pairwise comparisons. If normality was violated, the Kruskal–Wallis rank sum test was used, and Dunn pairwise tests with Bonferroni adjustment (FSA package) were performed after an overall significant result. F or H statistics and their *p* values are reported. Parametric outcomes are presented as means, non-parametric outcomes as medians, and post hoc group differences are marked with letter codes. Percentage changes relative to the non-degraded grassland are also provided. For parametric data, the percentages were computed from group means, whereas for non-parametric data they were derived from medians. A plus or minus sign denotes an increase or decrease, with values rounded to two decimal places. The soil quality index (SQI) was derived in three steps. First, variables whose higher values indicate poorer status, for example, electrical conductivity (EC), were transformed before standardisation to yield positive indicators. Second, the data were standardised to Z scores [37]. Third, indicator weights were determined objectively with the entropy weight method [38], and the final SQI was calculated as a weighted sum of the standardised variables [37].

**Figure 2:**
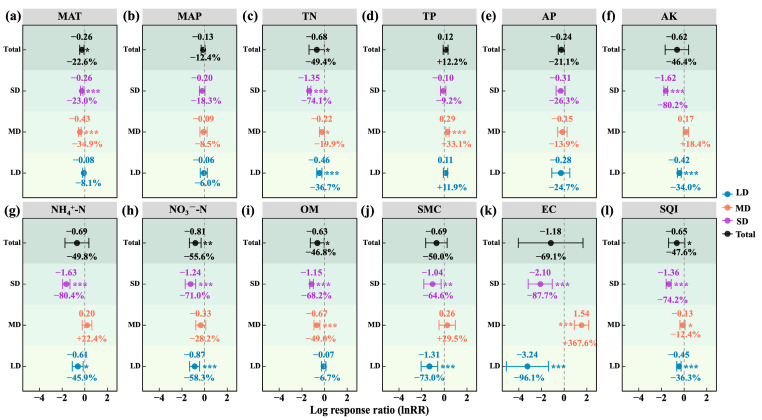
Climate and soil properties along a degradation gradient in alpine steppes. (**a**) MAT; mean annual temperature. (**b**) MAP; annual precipitation. (**c**) TN; total nitrogen. (**d**) TP; phosphorus. (**e**) AP; available phosphorus. (**f**) AK; available potassium. (**g**) NH4+-N; ammonium nitrogen. (**h**) NO3−-N; nitrate nitrogen. (**i**) OM; organic matter. (**j**) SMC; soil moisture content. (**k**) EC; electrical conductivity. (**l**) SQI; soil quality index. Note: The green background darkens with increasing degradation, from non-degraded to severely degradation alpine steppes. * 0.01 < *p* < 0.05; ** 0.001 < *p* < 0.01; *** *p* < 0.001.

Overall treatment effects relative to the non-degraded group were estimated within the log response ratio framework (lnRR, metafor package). A random-effects model fitted with REML was applied to the total dataset and to each degradation level, and point estimates together with their 95% confidence intervals and *p* values are reported (Figure 2). For ease of interpretation, lnRR values were back-transformed to ratios (degraded/non-degraded) and expressed as percentage differences, that is, (ratio − 1) × 100 percent [39].

Phyllosphere microbial sources were inferred with Source Tracker [40]. The alpha diversity of *S. purpurea* phyllosphere bacteria was quantified using richness, Shannon index and phylogenetic diversity (PD) (picante package) [41]. Group differences were then tested, and relationships with environmental factors were evaluated using Spearman correlations [42]. For beta diversity, Bray–Curtis distance matrices (vegan package) were computed and visualised through PCoA [43], and group differences were tested with PERMANOVA adjusted by the Bonferroni procedure for multiple comparisons [44]. To alleviate multicollinearity, variables with a variance inflation factor greater than ten were removed. The remaining predictors were linked to Bray–Curtis distances by distance-based redundancy analysis (dbRDA) [45], and the independent effects and significance of environmental variables were quantified through hierarchical partitioning [46].

Community mean tolerance breadth (CMTB) was calculated, with higher values denoting a greater proportion of taxa capable of withstanding broad environmental gradients. Community mean response asynchrony (CMRA) was also computed; larger CMRA values indicate more heterogeneous OTU-level responses and, consequently, broader community tolerance [47]. Both CMTB and CMRA were derived from 10 standardised soil variables and 2 standardised climatic variables, using a rarefaction depth equal to the total number of OTUs present in each sample and 999 random permutations to ensure metric stability [48]. Community resistance potential was evaluated with the resistance potential metric [48].

Key determinants of *S. purpurea* biomass were identified with random forest analysis (randomForest package) [49]; variable importance and significance were assessed with rfPermute, and overall model significance was evaluated with A3 [50]. The significant predictors selected by the random forest were subsequently regressed against biomass using linear models to confirm their direction and magnitude of effect. For these regressions, we assessed model assumptions by inspecting Q–Q plots and applying Shapiro–Wilk tests on residuals to check normality and by examining residuals vs. fitted plots to evaluate homoscedasticity; no strong violations were detected. All statistical analyses and data visualisation were conducted in R (version 4.4.1), unless otherwise specified.

## 3. Results

### 3.1. Changes in Habitat, Plant Community and Dominant Species Along the Degradation Gradient

As degradation intensity increased, marked declines relative to the non-degraded grassland became evident (Supplementary Table S2). At the overall effect level, mean annual temperature fell by 22.6%, total soil nitrogen by 49.4%, nitrate nitrogen by 55.6% soil organic matter by 46.8% and soil quality index (SQI) by 47.6% (Figure 2a,c,h,i,l; *p* < 0.05). Except for total soil phosphorus, all other measured variables exhibited overall effects below the non-degraded baseline.

**Figure 3:**
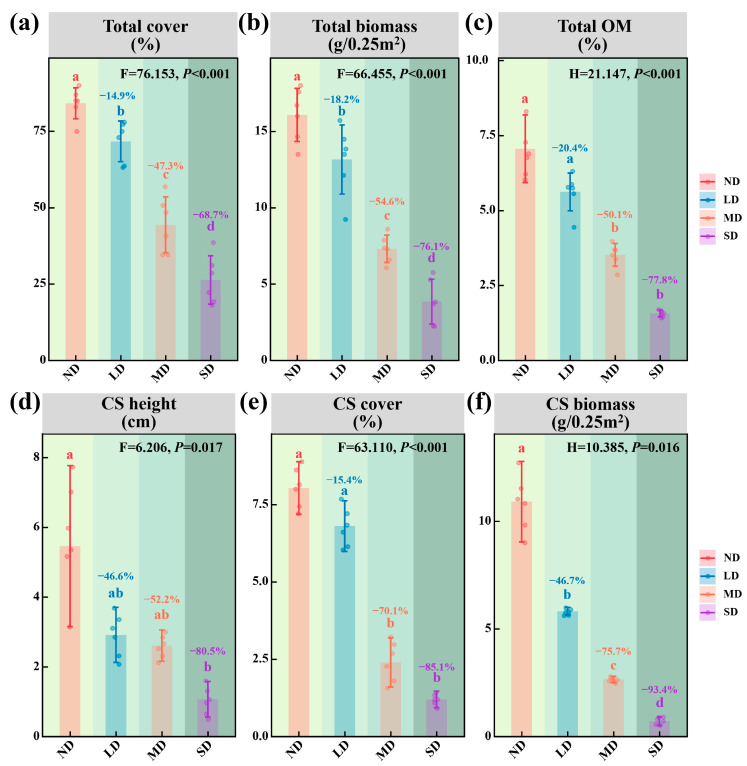
Community and dominant species along a degradation gradient in alpine steppes. (**a**) Total community cover. (**b**) Total community aboveground biomass. (**c**) Soil organic matter content. (**d**) Height of dominant species. (**e**) Cover of dominant species. (**f**) Aboveground biomass of dominant species. Note: Panels (**b**,**f**) report aboveground biomass. The green background darkens with increasing degradation, from non-degraded to severely degradation alpine steppes.

As degradation intensified, significant declines were detected in community-level metrics, with total vegetation cover, aboveground biomass and soil organic matter each falling markedly (Figure 3a–c; *p* < 0.001). Likewise, the dominant species Stipa purpurea displayed substantial declines in plant height, cover and aboveground biomass (Figure 3d–f; *p* < 0.05).

### 3.2. Phyllosphere Bacterial Diversity of Dominant Species Along the Degradation Gradient and Its Environmental Drivers

**Figure 4:**
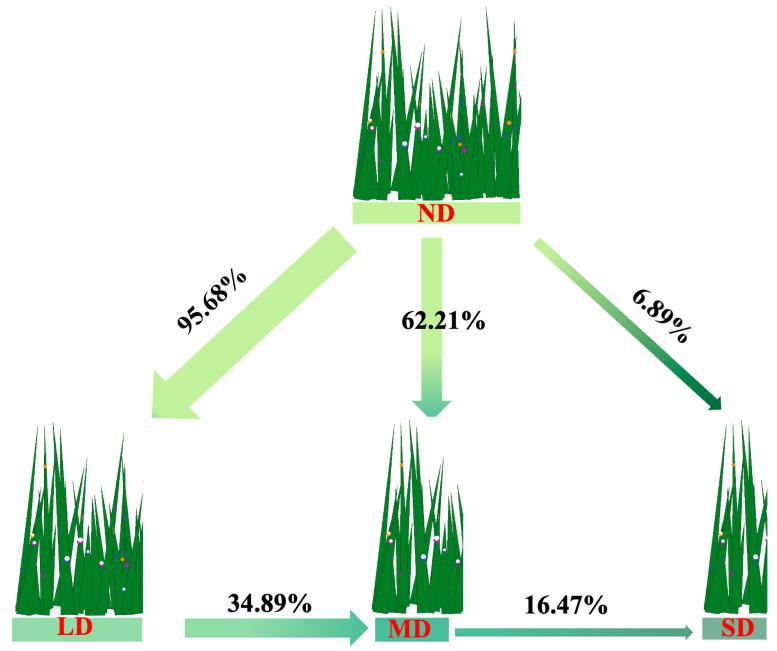
Source and sink patterns of phyllospheric bacterial communities of dominant species along a degradation gradient in alpine steppes. Note: Arrows start at the source and end at the sink, and arrow width is proportional to the relative contribution. Percent values indicate the share of each sink community that is attributed to a given source. The remaining fraction in each sink corresponds to the “unknown source” category in the Source Tracker analysis, that is, sequences not assigned to any of the four degradation stages included as explicit sources.

As degradation progressed, the fraction of dominant species’ phyllosphere bacterial communities inherited from the non-degraded (ND) stand declined steeply, falling from 95.68% in lightly degraded (LD) sites to 62.21% in moderately degraded (MD) and only 6.89% in severely degraded (SD) grasslands. Similarly, cross-level contributions decreased from LD to MD (34.89%) and from MD to SD (16.47%) (Figure 4). Overall, source inheritance declined stepwise along the degradation gradient, thereby indicating intensified community turnover and source drift.

**Figure 5:**
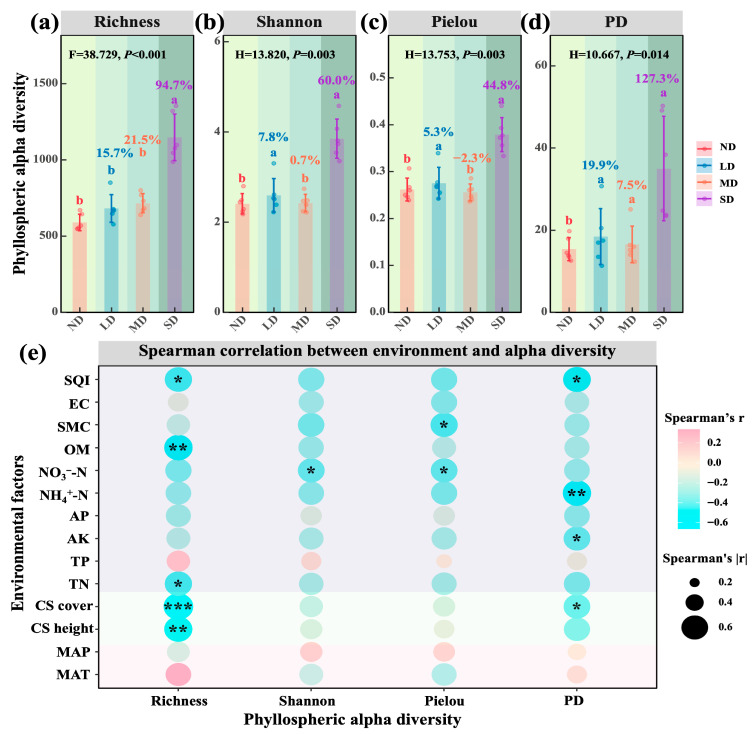
Phyllospheric bacterial alpha diversity of dominant species along a degradation gradient in alpine steppes and its determinants. (**a**) Richness. (**b**) Shannon index. (**c**) Pielou evenness. (**d**) PD; phylogenetic diversity. (**e**) Determinants of phyllospheric bacterial alpha diversity. Note: The green background darkens with increasing degradation, from non-degraded to severely degradation alpine steppes. * 0.01 < *p* < 0.05; ** 0.001 < *p* < 0.01; *** *p* < 0.001.

As degradation intensified, the phyllosphere bacterial richness of *Stipa purpurea* increased substantially (Figure 5a; *p* < 0.001). Conversely, Shannon diversity, Pielou evenness and phylogenetic diversity (PD) followed a significant non-monotonic pattern characterised by an increase, decrease and rise again along the gradient (Figure 5b–d; *p* < 0.05). Regarding driving factors (Figure 5e), richness was negatively influenced by Stipa height and cover, soil total nitrogen (TN), organic matter (OM) and soil quality index (SQI). Shannon diversity exhibited a significant negative correlation with nitrate nitrogen (NO_3_^−^ N), while Pielou evenness was negatively related to NO_3_^−^ N and soil moisture content (SMC), and PD was negatively associated with Stipa cover, available potassium (AK), ammonium nitrogen (NH_4_^+^ N) and SQI (*p* < 0.05).

**Figure 6:**
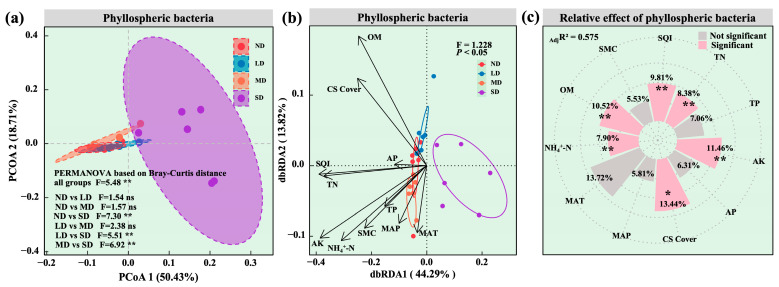
Phyllospheric bacterial community differences in dominant species along a degradation gradient in alpine steppes and their drivers. (**a**) Principal coordinates analysis (PCoA) of phyllospheric bacterial communities. (**b**) Distance-based redundancy analysis (dbRDA) of phyllospheric bacterial communities. (**c**) Key drivers of phyllospheric bacterial community change. * 0.01 < *p* < 0.05; ** 0.001 < *p* < 0.01.

Pronounced shifts in the phyllosphere bacterial community of the dominant species were observed along the degradation gradient. PERMANOVA confirmed significant differences among all groups (Figure 6a; F = 5.48, *p* < 0.01). Specifically, the community in the severely degraded grassland differed significantly from those in non-degraded (ND), lightly degraded (LD), and moderately degraded (MD) sites (ND vs. SD: F = 7.30, *p* < 0.01; LD vs. SD: F = 5.51, *p* < 0.01; MD vs. SD: F = 6.92, *p* < 0.01). After multicollinearity was eliminated, distance-based redundancy analysis indicated that *Stipa purpurea* cover (CS cover), MAT, MAP and soil variables together explained 44.29% of the variation on axis one (Figure 6b; F = 1.228, *p* < 0.05). Variation partitioning further showed that these factors collectively accounted for 57.5% of the total community variation (Figure 6c; _adj_R^2^ = 0.575). Among them, ammonium nitrogen (NH_4_^+^ N, 7.90%), total nitrogen (TN, 8.38%), SQI (9.81%), organic matter (OM, 10.52%), available potassium (AK, 11.46%) and CS cover (13.44%) made significant individual contributions.

### 3.3. Resistance of Dominant Species Phyllosphere Bacterial Communities Across Degradation Levels

**Figure 7:**
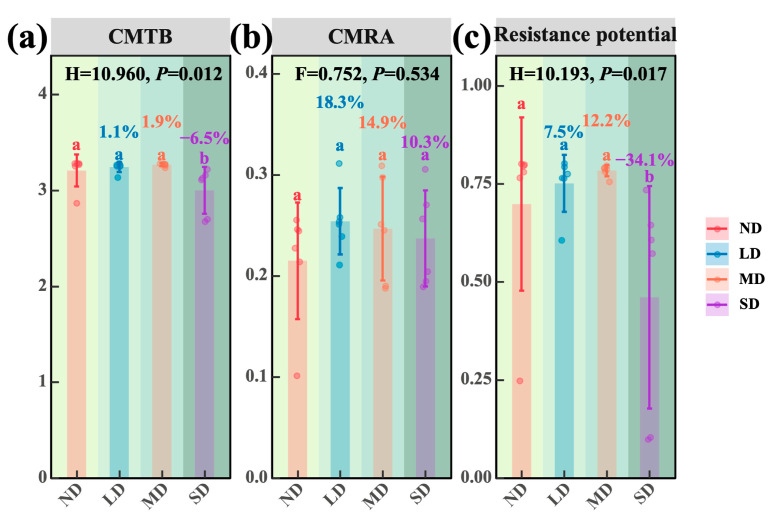
Resistance of phyllospheric bacterial communities of dominant species along a degradation gradient in alpine steppes. (**a**) CMTB; community mean tolerance breadth. (**b**) CMTA; community mean response asynchrony. (**c**) Resistance potential. Note: The green background darkens with increasing degradation, from non-degraded to severely degradation alpine steppes.

As degradation progressed, community-level indices of environmental tolerance and asynchrony exhibited divergent trajectories. Relative to the non-degraded grassland (ND), community mean tolerance breadth (CMTB) rose by 1.1 percent at the lightly degraded (LD) site and by 1.9% at the moderately degraded (MD) site, although neither change was significant; by contrast, CMTB declined significantly by 6.5% at the severely degraded (SD) site (Figure 7a; *p* < 0.05). For community mean response asynchrony (CMRA), LD, MD and SD showed increases of 18.3, 14.9 and 10.3%, respectively, compared with ND, yet none of these differences were significant (Figure 7b). Regarding resistance potential, LD and MD exhibited non-significant increases of 7.5 and 12.2%, whereas SD declined significantly by 34.1% (Figure 7c; *p* < 0.05). Taken together, light to moderate degradation exerted little influence on community asynchrony or resistance potential, whereas severe degradation caused a pronounced contraction of both tolerance breadth and resistance capacity.

### 3.4. Drivers of Dominant Species Biomass

**Figure 8:**
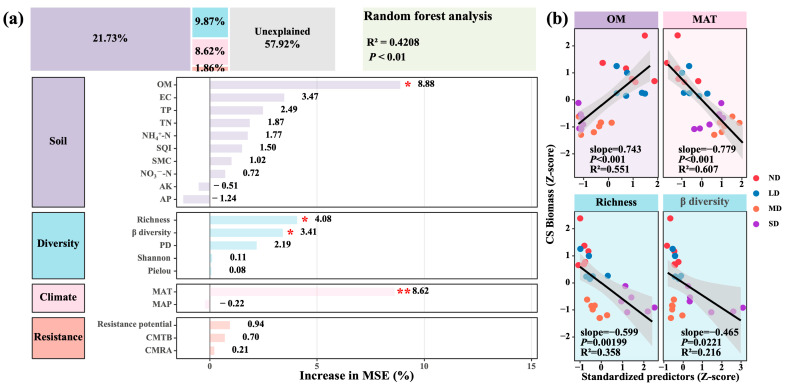
Determinants of biomass of dominant species in alpine steppes. (**a**) Random forest analysis of biomass determinants. (**b**) Correlations between key determinants and biomass. * 0.01 < *p* < 0.05; ** 0.001 < *p* < 0.01.

Across the degradation gradient from non-degraded to severely degraded grassland, climate, soil properties, phyllosphere bacterial diversity and community resistance together explained 42.08% of the variation in dominant species biomass (Figure 8a; R^2^ = 0.4208, *p* < 0.01). Individually, they contributed 8.62% (climate), 21.73% (soil), 9.87% (phyllosphere diversity) and 1.86% (resistance potential). Among these variables, soil organic matter (OM), species richness, β diversity and mean annual temperature (MAT) were significant predictors (*p* < 0.05). Correlation analyses of the significant variables identified by the random forest model confirmed the relationships: OM was positively correlated with dominant species biomass, whereas richness, beta diversity and MAT were negatively correlated (Figure 8b; *p* < 0.05). These findings further underscored the robustness of the random forest results. In summary, higher mean annual temperature and greater phyllosphere bacterial diversity (richness and β diversity) are associated with reduced biomass of the dominant species, whereas increased soil organic matter promotes biomass accumulation. This suggests that temperature and phyllosphere community structure exert suppressive influences on dominant species productivity, whereas organic matter exerts a promotive effect.

## 4. Discussion

### 4.1. Degradation Signatures in Habitats, Plant Communities and Dominant Species

As degradation progressed, alpine steppes experienced simultaneous declines in climatic and edaphic conditions that cascaded through the plant community to the dominant species. Compared with the non-degraded grassland, mean annual temperature decreased by 22.6%, indicating that degraded sites are characterised by an increasingly cold and dry microclimate, consistent with previous studies [51]. Soil total nitrogen and nitrate nitrogen declined by approximately 49.4 and 55.6%, respectively, whereas organic matter declined by 46.8% and soil quality index (SQI) by 47.6%, highlighting a concurrent deficiency of essential nutrients and carbon sources [2]. Notably, total phosphorus remained comparatively stable, suggesting that degradation is driven primarily by nitrogen and carbon cycling rather than by indiscriminate nutrient loss [52]. Beyond chemical properties, our WRB-based classification and particle-size data show that vegetation degradation is accompanied by a clear coarsening of soil texture. Non-degraded (ND) alpine steppes occur on Cryosols with a silt loam texture, lightly degraded (LD) steppes on loam soils, moderately degraded (MD) steppes on sandy loam soils and severely degraded (SD) steppes on loamy sand Regosols (Supplementary Table S1). This shift towards sandier, less structured soils likely reduces water-holding and nutrient-retention capacity and increases erosion risk, which is consistent with our observed declines in soil organic matter, nitrogen and soil quality index along the degradation gradient.

Vegetation level responses were equally striking: community cover and aboveground biomass declined significantly, and *Stipa purpurea*, the dominant species, exhibited marked reductions in height, cover and biomass. These losses not only weaken the grassland’s capacity to sequester carbon but also reduce its ecological barrier function [8]. Within alpine steppes, dominant species biomass correlates strongly and positively with community net primary productivity, making it a critical indicator of ecosystem degradation and restoration potential [53].

Taken together, our findings demonstrate that alpine steppe systems undergo parallel declines in water and heat supply, soil quality and vegetation productivity along the degradation gradient, underscoring their tight ecological coupling.

### 4.2. Nonlinear Responses of Dominant Species Phyllosphere Bacterial Diversity and Its Environmental Drivers

As degradation progressed, the dominant species’ phyllosphere bacterial community exhibited a clear pattern of source attenuation followed by accelerated turnover, paralleling the coupled degradation of habitat, community and species. Bacterial communities in lightly, moderately and severely degraded grasslands retained 95.68, 62.21 and 6.89% of the taxa present in non-degraded (ND) grassland, whereas carryover from LD to MD and from MD to SD declined to 34.89 and 16.47%. Consequently, increasing degradation diluted the historical imprint of the community and facilitated the immigration of novel taxa, leading to faster community renewal, a pattern consistent with reports of higher immigration rates and stronger environmental filtering in degraded alpine steppes [18]. For α diversity, species richness increased significantly with degradation (Figure 5a; *p* < 0.001), whereas the Shannon index, Pielou evenness and phylogenetic diversity (PD) followed a non-monotonic sequence of increase, decrease, and rise again (Figure 5b–d; *p* < 0.05). This finding mirrors studies on the Tibetan Plateau showing that a larger species pool does not necessarily coincide with higher evenness or phylogenetic breadth [17,54]. The higher phyllosphere bacterial richness observed under severe degradation therefore likely reflects a relaxation of host filtering and increased colonisation by opportunistic or generalist taxa from soil and atmospheric sources [55], consistent with an invasion-type mechanism under strong disturbance rather than a stabilising “intermediate disturbance” scenario [56]. Driver analysis indicated that richness was negatively associated with dominant species height and cover, soil total nitrogen, organic matter (OM) and SQI, implying that weaker host filtering and declining soil quality create phyllosphere niches for broad niche newcomers [57]. The Shannon and Pielou indices were negatively correlated with nitrate nitrogen and soil moisture content, underscoring the direct constraints imposed by mineral nitrogen form and water availability on community evenness [58]. PD displayed negative associations with host cover, available potassium, ammonium nitrogen and SQI, suggesting that accelerated turnover did not translate into greater phylogenetic breadth [59]. At the β diversity level, the severely degraded sites separated significantly from all others, and distance-based redundancy analysis demonstrated that host cover, mean annual temperature (MAT), mean annual precipitation (MAP) and soil nitrogen and carbon metrics jointly explained 57.5% of the variation. Among these variables, ammonium nitrogen, total nitrogen, SQI, OM and available potassium contributed in ascending order, underscoring a multifactor modulation by host traits, climate and soil [17,18].

Taken together, degradation influences the dominant species’ phyllosphere bacterial community through four intertwined pathways, weakened host filtering, soil impoverishment, shifts in nitrogen forms and altered hydrothermal regimes, and manifests as a diminished source legacy, elevated species richness, fluctuating evenness and phylogenetic diversity, and pronounced spatial restructuring of the community.

### 4.3. Differences in Resistance Metrics and Determinants of Dominant Species Biomass

As degradation intensified, CMTB increased by 1.1% in lightly degraded (LD) and 1.9% in moderately degraded (MD) grasslands relative to non-degraded grassland (ND), yet neither change was significant. Resistance potential likewise rose slightly (7.5 and 12.2%, not significant), and CMRA surpassed ND levels across all degraded classes (18.3 to 10.3%, not significant). We infer that at the LD to MD stages, host filtering on the dominant species’ phyllosphere bacterial community outweighs environmental filtering: source tracking revealed higher species turnover, increased richness and altered community structure (PCoA and PERMANOVA, *p* < 0.01), collectively broadening the community’s tolerance spectrum and latent resistance. Once severe degradation (SD) is reached, environmental filtering surpasses host selection. Ongoing resource depletion in climate and soil alters host traits (height, cover, biomass), accelerates bacterial turnover, increases diversity and restructures the community, yet CMTB and resistance potential are now significantly reduced (6.5 and 34.1%, respectively; Figure 7). Hence, along the alpine steppe degradation gradient, the balance between host and environmental filtering shifts markedly: LD to MD stages are host-driven, with broader tolerance breadths and higher latent resistance, whereas SD is environment-driven, causing host–microbe decoupling and a sharp decline in resistance potential. This mechanism accords with recent findings for alpine steppes on the Tibetan Plateau [60]. Hu et al. reported that grazing induced degradation diminished the stability of phyllosphere and rhizosphere microbial networks in Kobresia humilis, weakening host–microbe synergy [18]. Li et al. showed that, in clonal plants, host filtering maintains microbial resistance and functional coherence under stable resources, whereas intensified environmental filtering under resource scarcity accelerates turnover and reduces system resilience [61]. Similar patterns have been observed in alpine wetlands and grasslands, where aridification shifts assembly mechanisms and reduces community resistance [62].

In alpine steppes, biomass accumulation of the dominant species is co-regulated by climate, soil, phyllosphere microbial diversity and community resistance. These four factor groups jointly explained 42.08% of biomass variation (R^2^ = 0.4208, *p* < 0.01); soil contributed the most (21.73%), followed by phyllosphere diversity (9.87%) and climate (8.62%), whereas resistance accounted for only 1.86%. This hierarchy aligns with recent coupling studies of ecosystem structure and function on the Plateau [63,64]. Among individual variables, organic matter correlated positively with biomass, underscoring soil carbon supply as a primary driver of productivity in alpine steppes [49]. By contrast, phyllosphere bacterial richness and beta diversity were negatively correlated with biomass, echoing a high turnover and low synchrony response under chronic warming and drought, where diversity expansion failed to enhance host performance [65]. The negative influence of climate factors, particularly mean annual temperature (MAT), on biomass was likewise confirmed; elevated temperatures can reduce biomass accumulation by altering microbial functions and carbon cycling [66]. Under such conditions, warming is expected to intensify heat- and drought-induced stress in *Stipa purpurea*, leading to reduced stomatal conductance, constrained carbon assimilation and a higher proportion of assimilated carbon being consumed by maintenance respiration rather than biomass accumulation [67].

In sum, climate serves as a background driver, soil quality as the dominant tier, and phyllosphere diversity as a secondary tier, together shaping the weighting scheme that determines dominant species biomass in degraded alpine steppes. It should also be noted that all phyllosphere samples were collected in mid-summer, so our results represent a growing-season snapshot of community structure and assembly along the degradation gradient; seasonal changes in phyllosphere composition and function were not captured here and warrant multi-season sampling in future work.

## 5. Conclusions

Our findings reveal a multidimensional response pattern in alpine steppes along the degradation gradient. In these alpine steppes, vegetation degradation is accompanied by a shift in soils from finer-textured Cryosols to coarser-textured Regosols and by a decline in the soil quality index, while mean annual temperature increases and soil nitrogen and soil organic matter decrease; together, these changes drive reductions in community-level productivity and dominant-species biomass. Among all variables, soil organic matter emerges as the principal positive determinant of dominant species biomass, whereas higher mean annual temperature coupled with elevated phyllosphere bacterial richness and beta diversity suppress productivity. These findings suggest that, under ongoing global warming, effective management of alpine steppe degradation should prioritise the light to moderate stages. Specifically, interventions that (i) enhance soil quality and (ii) maintain the structural dominance of the dominant species, thereby retaining its phyllosphere microbial host, are essential for sustaining the long-term stability and sustainable utilisation of alpine steppes.

## Figures and Tables

**Table 1:** Geographic coordinates (latitude and longitude) and elevation of alpine steppe plots along the degradation gradient.

Degradation Degree	Longitude (°)	Latitude (°)	Altitude (masl)
ND (Non-Degradation)	98°25′57.9163″ E	38°52′20.7166″ N	3498.6
LD (Light Degradation)	98°21′13.4802″ E	38°53′08.6719″ N	3342.5
MD (Moderate Degradation)	98°17′45.6189″ E	38°55′18.6818″ N	3428.5
SD (Severe Degradation)	98°22′25.5231″ E	38°50′20.4025″ N	3296.8

## Data Availability

The raw sequence data from this study were deposited in the NCBI database with the study accession number PRJNA1348604 and are publicly accessible at https://ncbi.nlm.nih.gov/sra (accessed on 28 October 2025).

## Supplementary Materials

The following supporting information can be downloaded at: https://www.mdpi.com/article/10.3390/microorganisms13122787/s1, Table S1: Soil classification and texture along a degradation gradient in alpine steppe; Table S2: Soil physicochemical properties of alpine steppe soils under different degradation levels.
