# Supplementary material for: Mean Annual Temperature, Soil Organic Matter and Phyllospheric Bacterial Diversity Shape Biomass of Dominant Species Along a Degradation Gradient in Alpine Steppes: A Case Study from the Qinghai–Tibet Plateau
Source: Microorganisms. 2025 Dec 7;13(12):2787. doi: 10.3390/microorganisms13122787 (PMC12735518; doi:10.3390/microorganisms13122787)
Supplement: Supplementary file 1 [file microorganisms-13-02787-s001.zip › microorganisms-3984716-supplementary.pdf]

Supplementary materials for

Mean annual temperature, soil organic matter, and Phyllospheric bacterial diversity shape biomass of constructive species along a degradation gradient in alpine steppes: A case study from the Qinghai–Tibetan Plateau

Kaifu Zheng, Xin Jin, Jingjing Li, Guangxin Lu*

College of Agriculture and Animal Husbandry, Qinghai University, Xining 810016, China; zhengkf@qhu.edu.cn (K.Z.); 18894310895@163.com (X.J.); 1961507783@qq.com (J.L.)
* Correspondence: lugx74@163.com; Tel.: +86-13897216290

Table S1. Soil classification and texture along a degradation gradient in alpine steppe.

Degradation degree	Soil Group	Sand (%)	Silt (%)	Clay (%)	Texture Class
ND (Non Degradation)	Cryosol	20	60	20	Silt loam
LD (Lightly Degradation)	Cambisol	40	45	15	Loam
MD (Moderately Degradation)	Gleysol	60	30	10	Sandy loam
SD (Severely Degradation)	Regosol	80	15	5	Loamy sand

Note: Soil classification follows the international WRB 2022 system (IUSS Working Group WRB, 2022). All properties were measured on composite mineral topsoil samples collected from 0–20 cm depth. Sand, silt and clay are expressed as mass percentages and were used to define the corresponding soil texture classes.

Table S2. Soil physicochemical properties of alpine steppe soils under different degradation levels.

Variables	ND	LD	MD	SD
TP（mg/kg）	319.58±20.36b	357.46±25.67ab	425.48±18.15a	312.13±29.44b
TN（mg/kg）	2055.02±128.52a	1299.86±129.79c	1646.17±143.55b	511.02±23.16d
AP（mg/kg）	6.67±1.06a	5.02±1.88a	5.74±0.81a	4.69±0.63a
AK（mg/kg）	198.44±11.27a	130.91±8.41b	235.03±24.37a	40.31±2.48c
NH_4^+-N（mg/kg）	8.64±1.09a	4.67±0.10b	10.57±1.86a	1.79±0.18b
NO_3^--N（mg/kg）	32.31±7.01a	13.49±0.86bc	23.20±1.43ab	9.49±0.76c
OM（%）	7.06±0.35a	5.62±0.25a	3.52±0.24b	1.56±0.02c
SMC（%）	37.33±13.72a	10.09±0.61b	48.35±0.73a	11.32±1.70b
EC（us/cm）	0.94±0.29b	0.04±0.03c	4.37±0.43a	0.06±0.05c

TN; Total nitrogen. TP; phosphorus. AP; Available phosphorus. AK; Available potassi-um. NH_4^+-N; Ammonium nitrogen. NO_3^--N; Nitrate nitrogen. OM; Organic matter. SMC; Soil moisture content. EC; Electrical conductivity. ND; Non Degradation. LD; Lightly Degradation. MD; Moderately Degradation. SD; Severely Degradation. Note: Soil properties were measured in the 0–20 cm layer. Values are means ± SE (n = 6). Different lowercase letters within a row indicate significant differences among soil variables at $P < 0.05$ (one-way ANOVA followed by Tukey’s HSD test or appropriate alternatives).
